# Peripheral and Central Determinants of a Nociceptive Reaction: An Approach to Psychophysics in the Rat

**DOI:** 10.1371/journal.pone.0003125

**Published:** 2008-09-03

**Authors:** Jean-Michel Benoist, Ivanne Pincedé, Kay Ballantyne, Léon Plaghki, Daniel Le Bars

**Affiliations:** 1 INSERM, U-713, Paris, France; 2 Laboratoire de Neurophysiologie, Faculté de Médecine Pitié-Salpêtrière, Paris, France; 3 Université Pierre et Marie Curie, Faculté de Médecine Pitié-Salpêtrière, Paris, France; 4 The Dental School, University of Dundee, Dundee, Scotland; 5 Unité READ, Université catholique de Louvain, Brussels, Belgium; University of Sydney, Australia

## Abstract

**Background:**

The quantitative end-point for many behavioral tests of nociception is the reaction time, i.e. the time lapse between the beginning of the application of a stimulus, e.g. heat, and the evoked response. Since it is technically impossible to heat the skin instantaneously by conventional means, the question of the significance of the reaction time to radiant heat remains open. We developed a theoretical framework, a related experimental paradigm and a model to analyze in psychophysical terms the “tail-flick” responses of rats to random variations of noxious radiant heat.

**Methodology/Principal Findings:**

A CO_2_ laser was used to avoid the drawbacks associated with standard methods of thermal stimulation. Heating of the skin was recorded with an infrared camera and was stopped by the reaction of the animal. For the first time, we define and determine two key descriptors of the behavioral response, namely the behavioral threshold (Tβ) and the behavioral latency (Lβ). By employing more than one site of stimulation, the paradigm allows determination of the conduction velocity of the peripheral fibers that trigger the response (V) and an estimation of the latency (Ld) of the central decision-making process. Ld (∼130 ms) is unaffected by ambient or skin temperature changes that affect the behavioral threshold (∼42.2–44.9°C in the 20–30°C range), behavioral latency (<500 ms), and the conduction velocity of the peripheral fibers that trigger the response (∼0.35–0.76 m/s in the 20–30°C range). We propose a simple model that is verified experimentally and that computes the variations in the so-called “tail-flick latency” (TFL) caused by changes in either the power of the radiant heat source, the initial temperature of the skin, or the site of stimulation along the tail.

**Conclusions/Significance:**

This approach enables the behavioral determinations of latent psychophysical (Tβ, Lβ, Ld) and neurophysiological (V) variables that have been previously inaccessible with conventional methods. Such an approach satisfies the repeated requests for improving nociceptive tests and offers a potentially heuristic progress for studying nociceptive behavior on more firm physiological and psychophysical grounds. The validity of using a reaction time of a behavioral response to an increasing heat stimulus as a “pain index” is challenged. This is illustrated by the predicted temperature-dependent variations of the behavioral TFL elicited by spontaneous variations of the temperature of the tail for thermoregulation.

## Introduction

Pain is a subjective sensory and emotional experience, which can be estimated in non-communicating individuals, only by examining the reaction elicited by a presumed noxious stimulus (that is termed “nociceptive”). The quantitative primary end-point for many behavioral tests of nociception in animals is the reaction time (t_R_), i.e. the time lapse between the beginning of the application of a nociceptive stimulus and the evoked response. Its measurement is straightforward when short duration tightly time-locked stimuli are delivered, e.g. electrical stimuli. However, the situation is more complex when the stimulus intensity is gradually increased while the stimulus is being delivered. This is the case with thermal stimulation, which is the most frequently used in behavioral tests of nociception (e.g. the so-called “tail-flick” test) because it stimulates specific receptors, called nociceptors, in the skin [1]. Indeed, it is technically impossible to heat the skin instantaneously by conventional means: thermal stimulation is always progressive.

**Figure 1:**
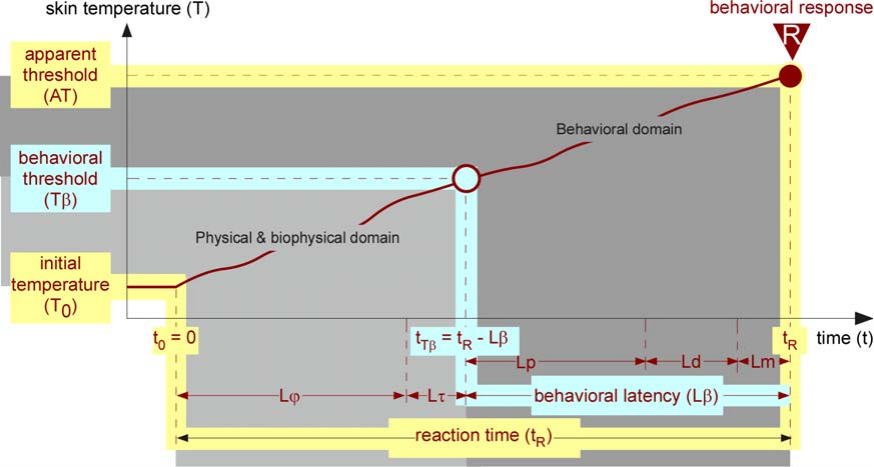
Theoretical analysis of a behavioral response to heating. In this and the forthcoming Figures, the measurable variables are indicated with an yellow background while variables to be determined are indicated with a blue background. Individual curves of interest are shown in brown. Symbols, Abbreviations and Units can be found in table 1. The behavioral response R results mainly from a serial processing along the dedicated pathways involving successive time epochs. As proposed by Luce [3], we will reserve the term latency (L) to an unobserved hypothetical time. Specifically, Lτ is the time required for heat to be transduced by nociceptors into neuronal spikes, which in turn are transmitted toward and received by the CNS. Lp is the transit time for these spikes to reach the CNS. Ld is the “decision” time required by the CNS for interpreting and processing this information for an order to be sent to the motor system (CDP). Lm is the time required for a motor response to be triggered. However, as it is physically impossible to heat the skin instantaneously, the reaction time t_R_ comprises an additional period of heating (Lϕ). Lϕ is completely dependent upon the heating rate and varies according to the experimental protocol. The other latencies are biological variables with Lτ≪Lp [11], [55], [56] and Lm≪Ld [57].

As any psychophysical process [2], [3], a nociceptive behavioral response results from a series of sequential events, each with its own duration. To clarify this, we shall start by dissecting the series of events that lead eventually to a nociceptive withdrawal reaction (R) in response to a heat stimulus (Fig. 1). The reaction time t_R_ is the sum of sequential physical (Lϕ), biophysical (Lτ) and behavioral (Lβ) latencies. Lϕ is the duration of the skin heating process starting at the initial skin temperature T_0_. Lτ is the transduction period, i.e. the time required for heat to be transduced by nociceptors into neuronal spikes. Lβ is the sum of: (1) the peripheral latency (Lp) required for the nociceptive information to reach the central nervous system (CNS) through the nerves; (2) the ‘decisional’ latency (Ld) required for central decision-making process (CDP), initiated by the arrival (and/or the accumulation) in the CNS, of a sufficient amount of nociceptive information to order the triggering of the withdrawal; and (3) the motor latency (Lm), time from motoneuron activation up to the shortening of the muscle. In other words, at the time when the ‘behavioral threshold’ (Tβ) level of thermal stimulation is achieved, i.e. time t_Tβ_ = t_R_−Lβ, the flow of information transmitted by the nociceptors is sufficient to trigger the behavioral response at time t_R_. Whether a response occurs depends on the state of the CDP occupying a time Ld. Tβ is defined by the occurrence of a behavioral response, itself depending on a go/no-go signal given by the CDP. Note that, the permissive state of the CDP is basically stochastic in nature and influenced by many factors, such as behavioral conditioning circumstances. We will use the term ‘apparent threshold’ (AT) for the skin temperature reached at the end of the sequence of latencies summing up to t_R_.

We aimed to develop an original paradigm to infer the latent psychophysical variables Lβ and Tβ, *a priori* unknown, from measurements of approachable quantities. To avoid the inconveniences of the conventional methods of thermal stimulation, a CO_2_ laser was used [4]. We report here experiments performed on the tail of the rat because, although it is an organ for thermoregulation and balance, it is widely used for the study of pain. However, the paradigm can be applied to any other mammal on any part of its body. In a first series of experiments, the basal temperature of the tail was stabilized at 34°C - i.e. the highest value recorded when the tail vessels were dilated (see Supporting Video S1). In a second series, the tail temperatures were extended to a physiological range of temperatures. Finally, we propose, and verified experimentally, a simple model for computing the variations of t_R_, the so-called ‘tail-flick latency’ (TFL), elicited by changes in either the power of the radiant heat source, the initial temperature of the skin or the site of stimulation along the tail.

## Results

### Behavioral threshold and behavioral latency

**Figure 2:**
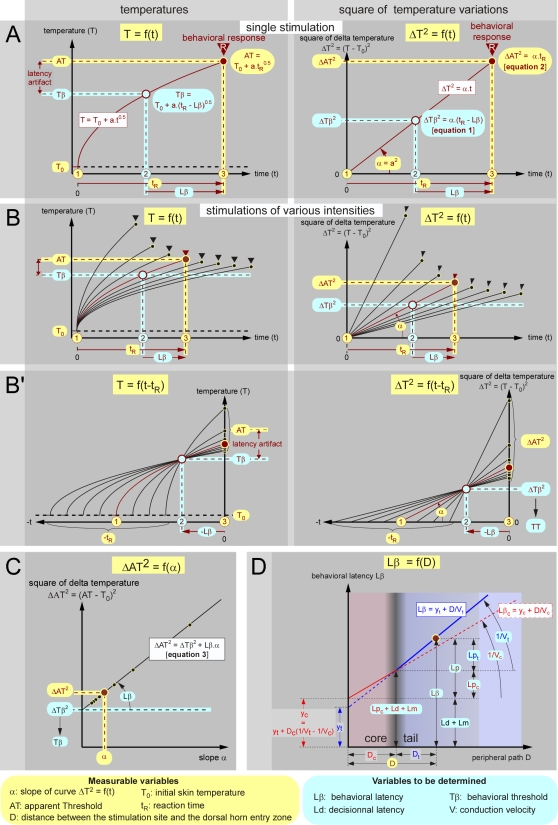
Theoretical analysis of a behavioral response to radiant heat. - A. When skin, at temperature T_0_, is exposed to a constant source of radiant heat, the temperature T increases with the square root of time (left graph). Expressed in terms of squared temperature variations, this relationship becomes linear (right graph). - B. If one varies the power source of radiation, a series of measures can be made, including the heating of the skin from the initial temperature T_0_ up to the apparent threshold AT (left graph). In terms of squared temperature variations, the relationships are linear and the constant term α can be calculated (right graph). This term reflects the density of power of the heating source. Overall, four quantities are potentially accessible to experimental measurements: T_0_, AT, t_R_ and α. - B′. One can modify the representation by adjusting the time scale of each individual curve for heating to the actual moment of the reaction. Such a change of origin allows one to visualize the back timing of events and to identify on the abscissa the point −Lβ and on the ordinate Tβ (left graph), or −Lβ and ΔTβ^2^ (right graph). Note that the latency artifact (AT–Tβ) increases with the stimulus intensity. - C. Relationship between the apparent threshold and the slope α. The intercept and the slope of this linear function represent ΔTβ^2^ and Lβ, respectively. - D. Theoretical relationship between the distance D, separating the site of stimulation on the tail from the dorsal root entry zone, and the behavioral latency Lβ. The available experimental data from the tail at T_0_°C are shown as a large blue line. The reciprocal of the slope of this line corresponds to the conduction velocity V_t_ of the fibers that triggered the reaction. However, the conduction velocity of these fibers increases when the coccygeal nerves travel through the core of the animal which is set at T_c_ 38°C by thermoregulatory processes. The two components of the peripheral process are shown in blue and red, respectively, with Lp = Lp_t_+Lp_c_ = D_t_/V_t_+D_c_/V_c_. The intercept y_c_ = y_t_+D_c_ (1/V_t_−1/V_c_) of the red straight line with the ordinate represents the part of Lβ that does not deal with the peripheral conduction, i.e. (Ld+Lm).

When the skin is exposed to a constant power source of infrared radiation, the temperature increases with the square root of time, according to the law of radiant heat transfer T = T_0_+a.t^0.5^ or, expressed in terms of temperature variation [5]: ΔT = T−T_0_ = a.t^0.5^ (Fig. 2A left). This quadratic relationship becomes linear in t by squaring the two terms of the equation: ΔT^2^ = a^2^.t = α.t (Fig. 2A right). Such temperature variations can be measured via a thermometric camera (see Fig. S1). Each behavioral trial can be summarized by four accessible quantities measured independently, namely the initial skin temperature T_0_, the apparent threshold AT, the reaction time t_R_ and the slope α. From the behavioral standpoint, the process of heating can be described by three key moments (Fig. 2A left):. the beginning of the stimulation, t = t_0_;. the moment of the triggering of the reaction defined by t = t_R_−Lβ and Tβ = T_0_+a.(t_R_−Lβ)^0.5^;. the moment of the reaction defined by t = t_R_ and AT = T_0_+a.t_R_^0.5^. Considering squared temperature variations, ΔT^2^ yields the following expressions (Fig. 2A right): ΔTβ^2^ = α.(t_R_−Lβ) [**equation 1**] and ΔAT^2^ = α.t_R_ [**equation 2**].

**Figure 3:**
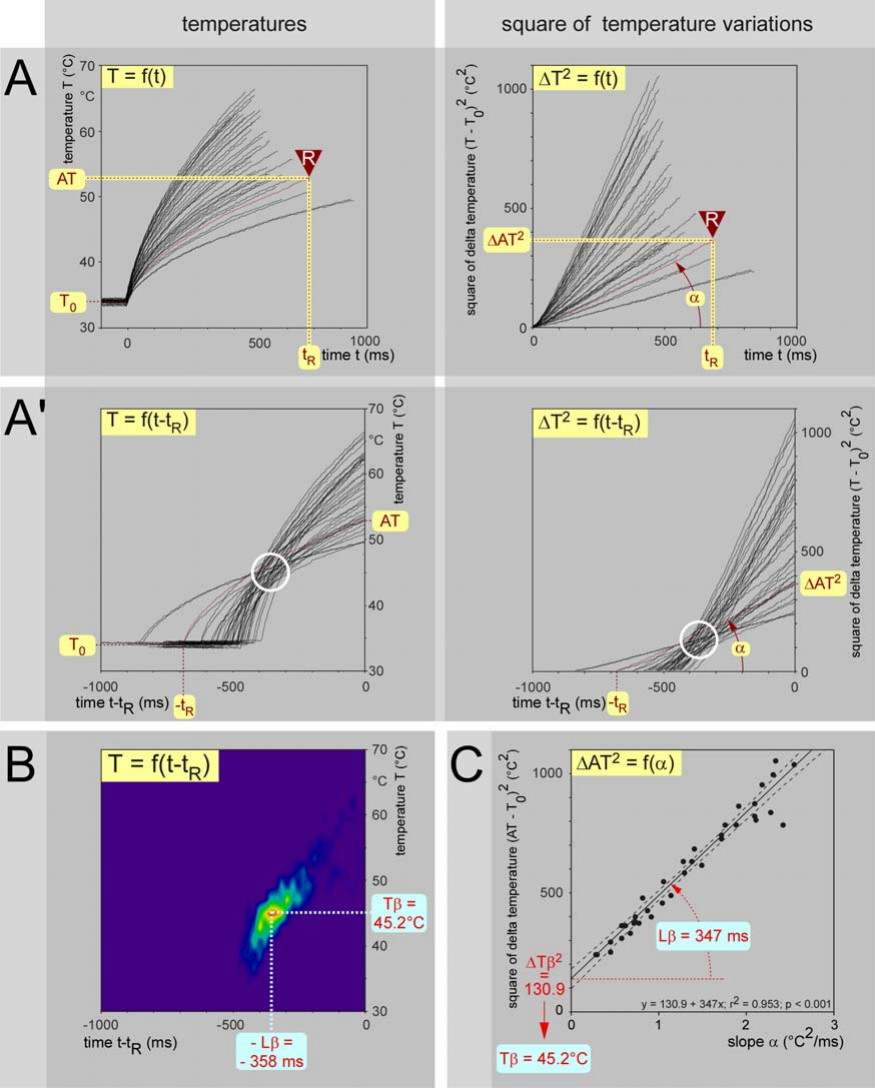
Analysis of behavioral responses elicited by stimulation of a given skin area. Example of behavioral responses of an individual animal, elicited by stimulation of the middle of the tail (distance D from this site to the entry zone in the cord = 200 mm). The stimulus (100–350 mW) was applied from time 0 until the movement of the tail. - A. Temporal evolution of the temperature of the skin (37 trials). Left graph: temporal evolution of the temperature of the skin. Right graph: Identical data expressed in terms of square of the difference of temperature. All these linear relationships were highly significant and their slopes could therefore be computed confidently. - A′. When one changes the origin to center the heating curves on the actual moment of the reaction, one can visualize the temporal evolution of the sequence of preceding events. Note the clear tendency of these curves to cross each other in a privileged zone (open white circle). - B. Analysis of the cluster of the intersections. The coordinates of the intersections of the curves shown in A′ (left graph) were analyzed in terms of relative frequency distribution. The false colors represent the relative density of intersections. The highest probability of density of intersections was found at coordinates −Lβ = −358 ms and Tβ = 45.2°C. - C. Relationship between the apparent threshold and the slope α, ΔAT^2^ = f(α). Such a plot gave rise to a highly significant linear relationship (dotted lines: ±95% CI). From the slope and intercept on the ordinate, one can then calculate Lβ = 347 ms and Tβ = T_0_+√ΔTβ^2^ = 45.2°C. Note that the methods shown in B and C provided almost identical results.

If T_0_ remains stable during the experimental procedure, we can infer Tβ the behavioral threshold temperature and Lβ the behavioral latency - which are presumably constant - from a series of trials where the power of the radiant heat source varies to produce an appropriate range of α (Fig. 2B & 3A). By adjusting the origin of the time scale of each individual heating curve to the actual time of the reaction, one can visualize the back-timing of events. In this temperature-time plane, each trajectory crosses every other (at a communal point) within a bounded region that allows determining Tβ and Lβ (Fig. 2B′ & 3A′).

Because of the stochastic nature of the psychophysical responses, the points of intersection of each curve with the others constituted a cluster in the temperature vs. time plot. Analyzing the cluster in terms of density of intersections revealed the highest density at coordinates Tβ and −Lβ (Fig. 3B). One can also substitute α.t_R_ of equation 2 in equation 1 thus obtaining after rearrangement: ΔAT^2^ = ΔTβ^2^+Lβ.α [**equation 3**] (Fig. 2C). When ΔAT^2^ was plotted as a function of α, a very strong linear relationship was observed; the slope and intercept of this relationship corresponded to Lβ and ΔTβ^2^, respectively (Fig. 3C). Thus, the paradigm and the mathematical processing of the data allow the determination of two key descriptors of the behavioral response to noxious heat, Tβ and Lβ, without any use of t_R_ - each individual response being fully described by T_0_, AT and α. Tβ and Lβ were both determined by the site of stimulation (i.e. by D, the distance between the stimulation site and the dorsal horn entry zone) and the temperature of the skin (i.e. by T_0_).

### Role of the stimulation site and conduction velocity of the fibers that triggered the reaction

**Figure 4:**
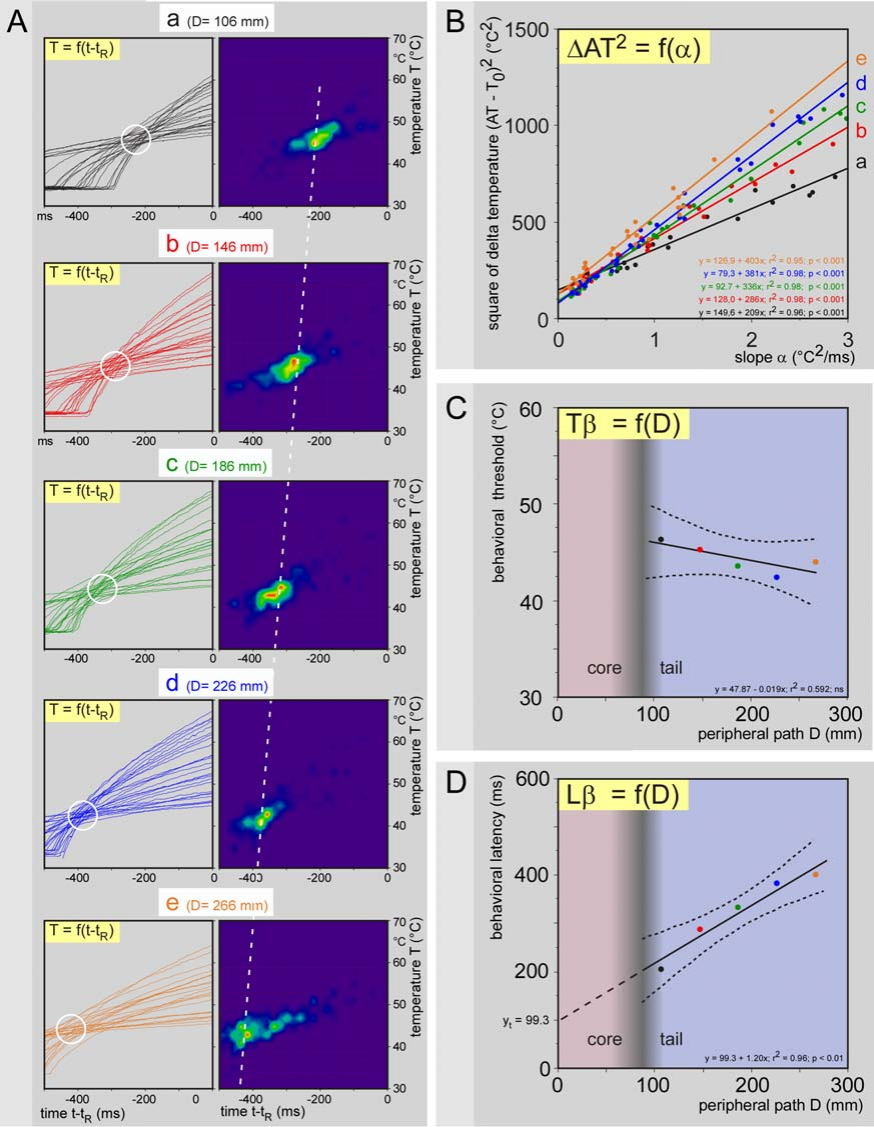
Analysis of the behavioral responses elicited by stimulation at several rostro-caudal levels on the tail. - A. Individual example of an analysis of the behavioral responses elicited by stimulation at 5 rostro-caudal levels on the tail, 40 mm apart (a–e from top to bottom). The left graphs show the temporal evolution of the temperature of the skin during the application of various powers of stimulation (100–350 mW) with the time origin being adjusted to the actual moment of triggering of the reaction. Note the clear tendency of these curves to cross each other in a privileged zone (open circles) and the progressive shift of this zone backward in time when the stimulation site moved from proximal to distal parts of the tail. The relative density of intersections is shown with false colors on the right panels. Note the shift of the coordinates of the highest probability of density of intersections (white dashed line). - B. Relationships between the apparent threshold AT and the slope α, ΔAT^2^ = f(α), calculated for the 5 sites of stimulation. The strong linear relationships provided accurate calculations of Tβ and Lβ for each level of stimulation. - C. Relationship between the calculated behavioral threshold Tβ and the distance D that separated the site of stimulation on the tail from the entry zone in the cord, as obtained from data shown in B (dotted lines: ±95% CI). - D. Relationship between the calculated behavioral latencies Lβ and the distance D that separated the site of stimulation on the tail from the entry zone in the cord, as obtained from data shown in B (dotted lines: ±95% CI). Lβ was directly proportional to D. The reciprocal of the slope represents the conduction velocity (0.83 m/s) of the fibers that triggered the reaction, in the part of their course within the tail. The decisional latency Ld can be estimated from the intercept y_t_ of the regression line Lβ = f(D) with the ordinate (Fig. 2D and see below).

**Figure 5:**
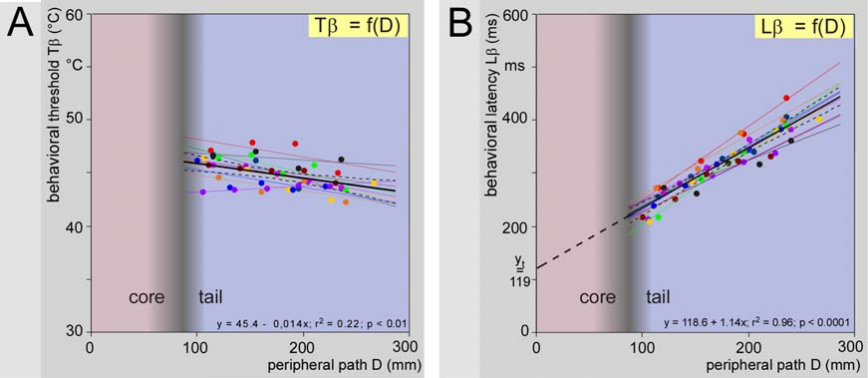
Overall influence of the levels of stimulation on behavioral thresholds and latencies. Observations were made on 10 rats with 4–5 stimulation sites (individual and overall regression lines are shown as fine and large lines, respectively). - A. Relationships between the calculated behavioral threshold Tβ and the distance D that separated the site of stimulation on the tail from the entry zone in the cord. On average, the behavioral threshold Tβ was 44.9 (44.5–45.3)°C with a significant tendency to decrease from the proximal to the distal parts of the tail, best described by the equation Tβ = 47.4−0.014 D (F_44,1_ = 12.2; p<0.01). The mean basal temperature of the skin T_0_ was 33.7±0.3°C. - B. Corresponding relationships between the calculated behavioral latencies Lβ and the distance D that separated the site of stimulation on the tail from the entry zone in the cord. Overall, there was a very significant linear relationship between D and Lβ (Lβ = 118.6+1.14 D; F_44,1_ = 271.7; p<0.0001). The decisional latency Ld can be estimated from the intercept y_t_ of the regression line Lβ = f(D) with the ordinate (Fig. 2E).

A classical method for calculating nerve conduction velocities was used by changing the distance D between the stimulation site on the tail and the dorsal horn entry zone in the spinal cord. The cluster referred to above moved to the left as D increased (Fig. 4A) and the ΔAT^2^ *vs.* α plots provided accurate calculations of Tβ and Lβ for each level of stimulation (Fig. 4B). On average, the behavioral threshold Tβ was 44.9 (44.3–45.7)°C, but displayed a tendency to get lower for distal sites of stimulation in the 42.5–46.4°C range (Fig. 4C & 5A). It is possible that the thickness of the epidermis along the tail decreases, thus facilitating the heat transfer to the nociceptors (see section “Measuring the temperature of the skin”). From a Darwinian perspective however, one can postulate that the extremities are more sensitive because of the survival value of protecting the more exposed parts of the body.

Lβ was directly proportional to D (Fig. 4D). The reciprocal of the slope represents the conduction velocity of the fibers that triggered the reaction, in the part of their course within the tail. The linearity and parallelism of the relationships Lβ = f(D) suggest homogeneity along the tail and across animals of the nerve fibers implicated in the reaction (Fig. 5B). The mean conduction velocity V_t_ (±95% CI) of these fibers in the section of their course traveling in the tail was 0.91 (0.81–1.01) m/s that fits with the 0.5–0.9 m/s of unmyelinated polymodal nociceptors recorded in the coccygeal nerve of the rat [6], [7]. We did not see any evidence that Aδ-fibers participate to the triggering of the tail withdrawal which confirms the conclusion of Danneman et al. [8]. Interestingly, Aδ-fibers recorded from the coccygeal nerve did not respond to radiant heat stimulation, at least up to 50°C [7] and, from a more general standpoint, the threshold of activation of individual Aδ-fiber nociceptors is higher than those for C-fiber nociceptors [9]–[11]. In any case, the possibility is now open for the non-invasive behavioral investigation of small primary afferent fibers, e.g. during the weeks or months periods of the full development of peripheral neuropathies such as those elicited by diabetes or chemotherapy [12].

### Ambient temperature as a key factor of variations

**Figure 6:**
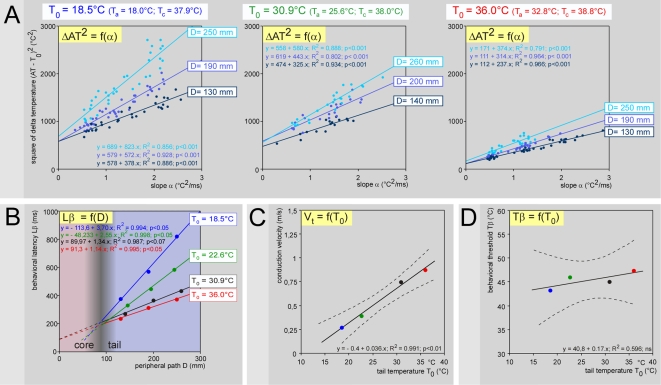
Effects of the ambient temperature on the behavioral responses: an example. - A. ΔAT^2^ = f(α) plots. The tail was stimulated at three rostro-caudal levels (dark to light blue curves; distance D that separated the site of stimulation on the tail from the entry zone in the cord is indicated on the right side of individual curves) in three different ambient temperatures that maintained the mean T_0_ at 18.5 (blue, left graph), 30.9 (green, middle graph) and 36.0°C (red, right graph). For a given skin temperature, the straight lines had close intercepts (ΔTβ^2^) but increasing gradients (Lβ) as the stimulation site moved distally. Both Lβ and ΔTβ^2^ fell as the ambient temperatures increased. The temporal evolution of the skin temperature of the corresponding individual heating curves can be seen in Fig. S2. - B. Lβ = f(D) plot. The conduction velocity accelerated dramatically as indicated by the drop of the slopes of the functions, when the temperature of the tail increased. Interestingly, the lines crossed near a point whose abscissa corresponded to the transition from the tail to the trunk (shaded area). - C. Corresponding V_t_ = f(T_0_) plot, revealing a Q_10_ = 2.12 between 20 and 30°C (dotted lines: ±95% CI). - D. Tβ = f(T_0_) plot. Note that the behavioral threshold Tβ increased as the ambient temperatures increased (dotted lines: ±95% CI).

The experiments described above were performed at room temperature with the tail skin temperature intentionally set at 34°C. We replicated the experiments with the rat being introduced in a chamber where the ambient temperature was maintained stable during a given session, but changed over sessions in the 17–34°C range. The tail was not intentionally heated and was stimulated at 3–4 rostro-caudal levels. For each basal skin temperature, the corresponding ΔAT^2^ = f(α) plots (Fig. 6A) allowed calculations of Lβ and Tβ for each level of stimulation on the tail. For a basal skin temperature, the straight lines had similar intercepts (ΔTβ^2^) but increasing slopes (Lβ), as one would expect as the stimulation site moved distally. Comparing the bundles of straight lines over increasing skin temperatures, revealed a fall in both Lβ and ΔTβ^2^. The slope of the Lβ = f(D) functions (Fig. 6B) fell as the tail temperature increased, which was testimony to increases in the conduction velocities of the fibers that triggered the behavioral reaction (Fig. 6C). Finally, the calculations revealed that the behavioral threshold Tβ increased slightly as the skin temperature T_0_ increased (Fig. 6D).

**Figure 7:**
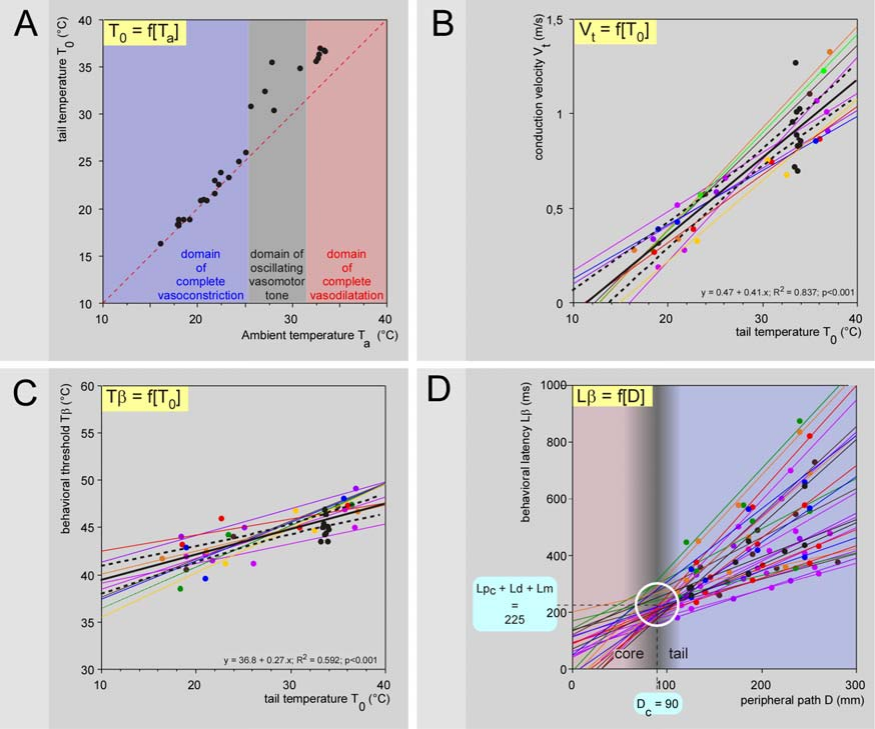
Effects of the ambient temperature on the behavioral responses: overall effects. - A. Effects of ambient temperature on the tail temperature. The red dashed line is the identity line. The temperature of the tail T_0_ was slightly above ambient temperature T_a_ for T_a_≤25°C (blue area) and several degrees above for T_a_≥32°C (red area); the core temperature T_c_ was significantly different between these two groups [37.7 (37.5–37.9) and 38.8 (38.4–39.3)°C, respectively; F_35,1_ = 28.9, P<0.001]. Between 25 and 32°C, it was essentially variable but the behavioral tests were performed during vasodilatation phases (grey area). - B. Individual V_t_ = f(T_0_) plots, obtained from 9 animals (fine lines). The overall V_t_ = f(T_0_) plot (large line) includes the data obtained in the experiments with T_0_ intentionally set at 34°C (black dots). A highly significant linear relationship was seen: V_t_ = −0.471+0.041.T_0_ (F_36,1_ = 185.5; p<0.0001). A mean Q_10_ = 2.3 (1.9–2.7) between 20 and 30°C was calculated (dotted lines: ±95% CI). - C. Corresponding individual and overall Tβ = f(T_0_) plots, as in B. A highly significant linear relationship was seen: Tβ = 36.8+0.27 T_0_ (F_36,1_ = 52.3; p<0.0001) (dotted lines: ±95% CI). D. Bundle of overall Lβ = f(D) straight lines obtained with various basal temperatures T_0_. These lines tends to cross each other in a privileged zone (white open circle) corresponding to the tail-trunk interface where the temperature of the nerves increases from T_0_ to the core temperature T_c_. Analyzing in terms of density the cluster of the intersection points of each straight line with the others in the D-Lβ plot revealed that the highest probability of intersections was located at coordinates D_c_ = 90 mm and (Lp_c_+Ld+Lm) = 225 ms, respectively. Incidentally, the time required for the nociceptive signals to travel within the core of the animal can be deduced: Lp_c_ = 99 (95–103) ms.

The overall results are summarized in figure 7. The temperature of the tail T_0_ was slightly above ambient temperature T_a_ when T_a_≤25°C, and several degrees above when T_a_≥30°C (Fig. 7A). Between 25 and 30°C, the basal temperature of the tail was essentially variable, the vasomotor tone of the tail oscillating over time between vasoconstriction and vasodilatation phases (see Supporting Video S1). The individual and overall relationships V_t_ = f(T_0_) revealed a linear function best described by the equation V_t_ = 0.041 T_0_−0.471, with Q_10_ between 20 and 30°C = 2.3 (1.9–2.7) (Fig. 7B). In the trunk, the corresponding conduction velocities of fibers at a core temperature T_c_ can then be easily deduced: V_c_ = V_t_+0.041 (T_c_−T_0_); on average, V_c_ = 1.1 (1.0–1.2) m/s for a 38.1 (37.9–38.3)°C mean T_c_. The dependency of the conduction velocity of peripheral fibers on temperature is a classical electrophysiological notion [13], [14]. During recordings of C-fibers in anesthetized cats, Q_10_ = 1.8 and 2.1 were reported from the saphenous and aortic nerves, respectively [15], [16], close to the value determined behaviorally in the present study. We shall give an idea of the tremendous variations introduced by this factor by noting the 2.7 times increase in the conduction velocity from 20 to 34°C - temperatures achieved in the rat tail in an air-conditioned room during vasoconstriction and vasodilatation, respectively.

The behavioral temperature threshold Tβ exhibited a large inter-individual variability with a significant tendency to increase with the basal tail temperature, best described by the equation Tβ = 0.27 T_0_+36.8 (Fig. 7C). This is not trivial, as the behavioral threshold increased by 4°C between 20 and 34°C. This finding might be interpreted as resulting from a CNS build up process resulting from population coding. Indeed, if one considers the peripheral information emanating from the tail - which makes sense as the tail is obviously a specific organ that one can consider as a functional entity - one sees a huge imbalance between information from the tiny heated site (∼9 mm^2^) and the surrounding non-stimulated areas (tail ∼5000 mm^2^). Such imbalance is indisputably reflected in the firing of the corresponding populations of dorsal horn neurons, which means that the thermal picture of the tail received by the brain is more or less contrasted according to the basal temperature. Low background temperatures facilitate the detection of a nociceptive event - thus lowering the pain threshold -, while higher background temperatures blur the detection of a nociceptive event - thus increasing the pain threshold.

Such mechanisms could explain the tendency of the behavioral threshold to be lower for distal sites of stimulation (see above). During the experiments described in figures 4C & 5A, the temperature of a given site was carefully adjusted for each stimulation, but not during the inter-stimulus intervals. Knowing that the proximal areas are always warmer than the distal parts of the tail, it follows that during the total duration of an experiment, there was a rough gradient of temperature over the tail during the inter-stimulus periods. Such a gradient could have influenced the determination of threshold, although the experimenter eliminated it at that very moment. These hypotheses, which suggest that the recent history of the thermal status of the tail did influence a later measure, obviously need further investigations.

There is no reason to believe that the behavioral threshold corresponds to the mean threshold of primary nociceptors [17]. We are dealing here with a threshold corresponding to the amount of nociceptive information that is sufficient for triggering the reaction. This “psychophysical” threshold integrates both peripheral and central mechanisms of nociception and has therefore to be higher than the mean thresholds of individual nociceptors. In this respect, the mean thresholds for activation of nociceptors in the tail, the firing of sacro-coccygeal dorsal horn neurons and tail withdrawals elicited by immersion of the tail in a water bath, have been reported to be 40.0, 42.2 and 43.7°C, respectively in anesthetized rats [18], [19]. This ranking exemplifies the increased level of convergence and summation needed to trigger neuronal events when one moves up the increasing complexity of the hierarchy of the nociceptive system. In spite of the wide range of thresholds (35–55°C) of single unmyelinated polymodal nociceptors in the coccygeal nerve [6], [19], the global output of the peripheral system appears sufficiently reproducible to generate stable behavioral responses.

Using conventional radiant heat, thresholds in the 40–43°C range were reported [20], [21], but the skin temperature was not measured in these studies. The observations that the (apparent) threshold and the magnitude of the tail-flick reflex increased when the power increased [22], [23] fit the present proposal that a withdrawal is triggered following a delay Lβ once the threshold Tβ is reached. During this period, the temperature continues to grow and activates nociceptors increasingly. The integral of the variation of temperature (AT−Tβ) during the period Lβ will determines the “total amount of nociceptive information” generated by the nociceptors that elicits the strength of the response. Thus, the steeper the heating slope, the stronger will be the response with a shorter reaction time. In this respect, it is interesting to note that the tail-flick is classically described as a brief movement of the tail, with the reaction time being shorter [20]–[22], [24]–[27] and the movement more vigorous [21], [28] when the intensity of the source of radiant heat increases.

In addition, the reported thresholds were in fact what we call here the apparent thresholds, dependent upon the steepness of the heat ramp. Tsuruoka et al. [23] recorded the electromyogram of tail muscles as an indicator of the magnitude of the tail-flick reflex and measured the (apparent) threshold elicited by radiant heat on the blackened tail. They found that from a well-defined baseline this threshold was around 42°C for the lowest power beam; this increased when the power increased (see also [22]). In this respect, we can define the difference (AT−Tβ) as the “latency artifact” (See Fig. 1A) because this is an obvious cause of variability in the determination of thermal thresholds. This was exemplified elegantly by the studies in humans by Yarnitsky and Ochoa [39], [30]. They showed that the method of limits often used for the determination of thermal thresholds in which the stimulus is stopped by the subject, resulted in greater overestimations of threshold when temperature rose faster. By comparison, the method of levels, where the subject's response does not influence the stimulus duration, produced identical thresholds whatever the rate of temperature variations. The notions of (“true”) behavioral and apparent thresholds developed here in an animal study are fully compatible with these observations in man.

### Decisional latency

The availability of a bundle of Lβ = f(D) relationships with a large range of slopes presented an opportunity for the estimation of further, important, psychophysical variables. The first step was to estimate the length of the peripheral nerves where the conduction velocity increased (given that there would be a higher core temperature within the animal). The V_t_ to V_c_ (conduction velocity of the fibers that triggered the reaction in the part of their course within the core) change would occur approximately at the tail-trunk interface, but it can be determined statistically (D_c_ = 90 mm) by considering the overall cluster of the intersection points of the straight lines Lβ = f(D), obtained from different tail temperatures T_0_ (Fig. 7D). The availability of both V_c_ and D_c_ provides keys for an estimation of the decisional latency Ld to be inferred from the intercept y_c_ of the regression line Lβ_c_ = f(D) with the ordinate: y_c_ = Ld+Lm = y_t_+D_c_(1/V_t_−1/V_c_) (See Fig. 2D). Since Lm = 4 ms for the tail-flick response in rats [8], on average, Ld = 132 (117–146) ms. Importantly, it was confirmed that Ld was not influenced significantly by T_0_. During this short period, modulation processes, notably those from supraspinal origin, have the opportunity to modify the withdrawal response.

We provided evidence for the homogeneity of V along the tail and across animals, when T_0_ is set at 34°C. We did correct the calculation of Ld by taking into account the increased temperature of the nerve in the core. A further cause of rise in conduction velocity, albeit probably minor, was neglected because of uncertainty: the possible confluence of peripheral fibers that gives rise to larger common branches [31], [32]. Increased conduction velocity was reported 2–3 cm before reaching the L4–L5 dorsal root ganglion neurons [33], [34]. Such uncontrolled factors of variations could add a few ms to the uncertainty of the estimation.

### Modeling and simulation of the ‘tail-flick latency’

**Figure 8:**
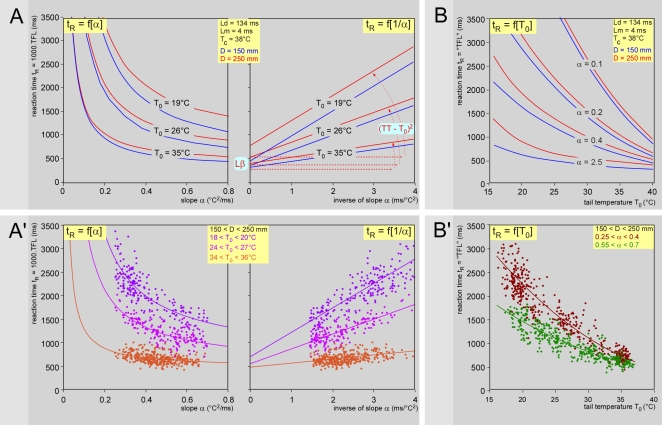
Modeling and simulation of the tail-flick “latency”. Results of simulations of the variations in the TFL introduced by varying the power of a radiant heat source: t_R_ = ΔTβ^2^/α+Lβ = (Tβ−T_0_)^2^/α+Lp+Ld+Lm = (Tβ−T_0_)^2^/α+D_t_/V_t_+D_c_/V_c_+Ld+Lm = (Tβ−T_0_)^2^/α+(D−90)/V_t_+90/V_c_+Ld+Lm. Taking into account the relation described above between temperature and (1) the conduction velocity of the fibers (V = 0.041 T−0.47) and (2) the behavioral threshold (Tβ = 0.27 T_0_+36.8), one can obtain, after rearrangement: t_R_ = (36.8−0.73 T_0_)^2^/α+90/(0.041 T_c_−0.47)+Ld+Lm+(D−90)/(0.041 T_0_−0.47). The numerical values of the parameters used in the equation are shown in the inserts. Upper graphs (A, B): theoretical curves; the blue and red lines are computations for stimulation of two sites on the tail spaced from the dorsal root entry zone by 150 and 250 mm, respectively. Lower graphs (A′, B′): corresponding experimental data obtained from six rats. - A. Role of heating power (α varying): theoretical curves. As expected from the form of the equation, the t_R_ = f(α) computation produced hyperbolas, with the horizontal asymptote representing Lβ (left graph). The right graph shows the relation t_R_ = f(α^−1^), which transforms this curve into a linear relationship. The slope and the intercept with the ordinate of the straight line represent (Tβ−T_0_)^2^ and Lβ respectively. These computations were made for three temperature of the tail: 19, 26 and 35°C, the latter being achieved when the animal dissipates heat by vasodilatation of the tail for any reason. - B. Role of basal skin temperature (T_0_ varying): theoretical curves. - A′. Role of heating power (α varying): experimental data and corresponding regression lines obtained from 6 animals are presented for three ranges of skin temperatures T_0_, namely 18–20 (violet), 24–27°C (mallow) and 34–36°C (orange). The stimulation power remained within a very limited range (16–20 mW) over a wide range of ambient temperatures giving rise to the following respective regressions lines: t_R_ = 702+521 (1/α); r^2^ = 0.681; p<0.001 and t_R_ = 553+317 (1/α); r^2^ = 0.585; p<0.001 and t_R_ = 485+84 (1/α); r^2^ = 0.165; p<0.001. - B′. Role of basal skin temperature (T_0_ varying): experimental data and corresponding regression lines from the same animals are presented for two ranges of heating rate expressed as α, namely 0.25–0.4 (deep green) and 0.55–0.7 (light colored green). Given that D = 200 mm, Ld = 134 ms, Lm = 4 ms and T_c_ = 38°C, we obtain after substituting and rearrangement of equation t_R_ = f[T_0_]: t_R_ = 0.53 T_0_^2^/α−53.7 T_0_/α+2683/(T_0_−11.46)+1354/α+221. The parameters and coefficients of regression were then estimated by a nonlinear least squares fit to the data: t_R_ = 2.53 T_0_^2^−240 T_0_+6023+82.2/(T_0_−21.3); r^2^ = 0.80; p<0.01 and t_R_ = 1.55 T_0_^2^−141 T_0_+3656−6.17/(T_0_−24.9); r^2^ = 0.72; p<0.01 for α = 0.25–0.4 and 0.55–0.7, respectively.

The possibility is open of computing the variations of the reaction time t_R_ (e.g. the so-called ‘tail-flick latency’, TFL), elicited by changing any of the parameters. In the classical tail-flick test, the principal source of variation introduced by experimenters is the power of heating of the electrical bulb used for achieving a predetermined range of TFL values - generally 2–4 seconds [35]. This corresponds here to variations in parameter α. As expected from the relation t_R_ = ΔTβ^2^/α+Lβ, such computation produced hyperbolae when the basal temperature is stable (Fig. 8A, left graph), with the horizontal asymptote representing Lβ. A simple anamorphous transformation [t_R_ = f(α^−1^)] linearized this relationship (Fig. 8A, right graph). The slope and the intercept with the ordinate of the straight line represent (Tβ−T_0_)^2^ and Lβ, respectively. The latter is the reaction time that one would expect following instantaneous heating (α→∞). Note the low impact of the stimulation site (or peripheral conduction distance) and the very high impact of the basal skin temperature (Fig. 8B). These theoretical relationships were validated in another series of experiments where the stimulation laser power remained within a very limited range (16–20 mW) over a wide range of ambient temperatures (Fig. 8A′ & 8B′). In other words, the predictive model of t_R_ was fully verified following variations of the radiant heat source or the basal temperature of the skin.

Incidentally, the predictive model of t_R_ offers an explanation for the surprising negative correlations between the rostro-caudal position of the stimulation site and the TFL even though the pathway for the afferent signals increases [36], [37]. This a priori paradoxical property was fully verified here by setting the parameters of the model to the values determined in the present study (see Fig. S3).

## Discussion

We have developed a psychophysical approach of nociceptive reactions, based on the joint analysis of the stimulus and the response and on the measurement of three observable variables, namely the initial temperature T_0_, the apparent threshold AT and the heating rate expressed as α. This paradigm allows one to reach the two key descriptors of the behavioral response to noxious heat in psychophysical terms without any use of the reaction time t_R_: the behavioral threshold Tβ and the behavioral latency Lβ, both of which are latent variables that are inaccessible with conventional methods. In addition, we calculated the conduction velocity of the peripheral fibers that trigger the reaction and proposed an estimate of the central decisional latency Ld, the most interesting part of Lβ to be investigated [3]. The usefulness of such an approach was demonstrated by providing new fundamental findings: the skin temperature, itself dependent on ambient temperature, very markedly influenced the behavioral threshold, the behavioral latency and the conduction velocity, but not the latency of the central decision-making process. Finally, we proposed and verified experimentally a simple model for computing the variations of t_R_, the so-called ‘tail-flick latency’ (TFL), elicited by changes in either the power of the radiant heat source, the initial temperature of the skin or the site of stimulation along the tail.

**Figure 9:**
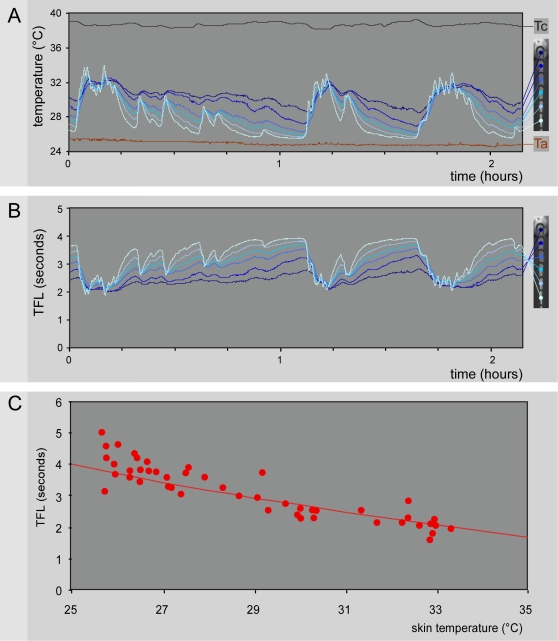
Spontaneous variations of tail temperature influences tail flick latencies. - A. Thermographic film of the tail of a rat placed in a conventional restrainer, recorded with a 320×240 pixels resolution at 1 Hz during 135 minutes (see Supporting Video S1). Steel rings maintained its tail (picture to the right with base of tail up and tip of tail down). From left to right temporal evolution of ambient temperature (Ta, lower brown trace), central core temperature (Tc, upper black trace) and tail temperatures recorded from base to tip at six equally distant places (blue dots and corresponding traces). Note the phases of vasoconstriction and vasodilatation throughout the recording session. In the most distal part of the tail, temperature varied by 8.5°C. - B. By using the model equation presented in Figure 8, and the temporal variations of T_0_ and T_C_ shown in panel A, tail flick latencies (TFL) were computed (Ld = 134 ms; Lm = 4 ms; α = 0.1). Simulated TFLs obtained for the most distal part of the tail ranged from 1.9 to 3.9 seconds. - C. Four rats were placed in the same conditions as for the recordings of the thermographic film in panel A, except that their tail was free for a behavioral response to be elicited. Only responses triggered by heating ramps characterized by slopes of 0.09<α<0.11 were considered (red dots). The red line represents the t_R_ = f(D) relationship for a single distal site of stimulation (D = 265 mm). Note that the behavioral TFL exhibited the temperature-dependent variations in the predicted range.

In our experiments, the behavioral latency was always less than 500 ms. The remaining part of the reaction time t_R_ mainly represents the time for the heating process Lϕ and, to a negligible extent, the transduction time Lτ required to achieve the behavioral threshold of the reaction Tβ. Lτ can be inferred from isolated primary afferent neuron recordings: Cesare and McNaughton [38] reported a 35 ms time for half activation of the inward current elicited by pulses of noxious heat. The sum t_R_ = (Lϕ+Lτ+Lβ corresponds to the “latency” usually observed in the conventional tail-flick test. In our experiments, the duration of heating was in the 0.25–2.5 seconds range (limited for technical reasons, see methods), whereas the current literature puts it in the 2–4 seconds range [35]. This undoubtedly means that most of this period is devoted to Lϕ, a simple physical process. This part increases when the reaction time increases by lowering either the power of the radiant heat source or the basal temperature of the skin. If t_R_ is the only measured end-point elicited by a given power of the source, there is no way of knowing whether any variation was produced by changes of either T_0_ or Tβ or both. The test validity, i.e. the degree to which a test actually measures what it purports to measure, is undoubtedly one of the most difficult problems to resolve in pain research [39]. The question of the validity of using a reaction time of a behavioral response to an increasing heat stimulus as a “pain index” is challenged. To illustrate this statement, we will consider the spontaneous variations of the temperature of the tail of a rat placed in a conventional restrainer (See Supporting Video S1). The temporal evolution of the skin temperature recorded along the tail (Fig. 9A) revealed phases of vasoconstriction and vasodilatation throughout the 2 hours recording session, with large amplitude of variations (25.4–33.9°C) for the most distal parts of the tail. Figure 9B used the predictive model of t_R_ to infer the corresponding predicted variations of the tail flick “latency”, elicited from rostro-caudal levels on the tail identical to the temperature recording sites. Note the ∼2 seconds range of variation for predicted TFL elicited from the tip of the tail. Measured in an identical experimental situation, behavioral TFL exhibited the predicted temperature-dependent variations (Fig. 9B).

In summary, we provide here both a theoretical framework and an experimental paradigm, based mainly on random variations of the stimulus, which for the first time enable the behavioral determinations of latent psychophysical (Tβ, Lβ, Ld) and neurophysiological (V_t_) variables that have been inaccessible with conventional methods. We believe that such an approach satisfies the repeated requests for improving nociceptive tests [35], [40] and offers a potentially heuristic progress for studying nociceptive behavior on more firm physiological and psychophysical grounds.

## Methods

### Animals

Experiments were performed on adult male Sprague-Dawley rats (Janvier, France) weighing 275–325 g in accordance with the National Institute of Health's ‘Guide for the care and use of Laboratory animals’, the European Communities Council Directive 86/609/EEC regulating animal research, and the ethics committee of the International Association for the Study of Pain [41], [42]. Each animal was placed in a Plexiglas device composed of a parallelepiped tank (L = 20 cm, l = 6 cm, H = 25 cm) open at its upper and lower extremities in order to ventilate the animal and allow accessibility to the body regions to be stimulated. The tank was suspended a few millimeters above the surface on which the rat was placed, ensuring sufficient space for the passage of the tail. A cylinder, which could slide along the tank, made it possible to adjust the device to the size of the rat. The longitudinal insertion of removable Plexiglas stems prevented the animal from standing-up.

The tail was depilated by means of a cream (Vichy®). Then the animal was habituated to this environment for one hour before the experiment. A colonic thermocouple was inserted 4 cm beyond the anal sphincter and fixed to the tank with adhesive tape. During the course of testing, stimulation was never applied during any behavior or postural adjustment of the animal.

At the end of the experiment, the animal was sacrificed by an overdose of pentobarbital and autopsied. The L3 vertebra was carefully identified and the distances (D) between the stimulation sites and this vertebra were measured. This level was considered as the main entry zone in the cord for afferent signals from the tail as: (1) the four major nerves innervating the tail, namely the dorsolateral and ventrolateral tail nerves, project to dorsal horn superficial laminae of S2-Co2 segments [43]; (2) these segments are located within vertebrae L2–L4 [44]; (3) the maximum N-wave dorsum potential elicited by electrical stimulation of the tail is found in the middle of a laminectomy of vertebrae L2–L4 [19], [45].

### Monitoring of the basal temperature of the tail

The tail, as a source of radiant heat, is an important organ for thermoregulation in the rat [46]. In the course of an experiment, the temperature of the tail varies spontaneously by several degrees (see Supporting Video S1). Since our approach was based on the measurement of the rise in temperature, it required the stability of the reference temperature of the zone being stimulated. We used an external device, consisting of a low power infrared lamp (50 W) coupled to a rheostat, to stabilize the temperature at 34±1°C. This temperature is the highest spontaneously achieved value we recorded when the tail vessels were dilated. In some experiments, the rat was introduced into a chamber where, during a given session, the ambient temperature was maintained stable in the 17–34°C range without any other additional source of heating.

### The stimulus

We set aside the use of light bulbs because they give emissions in the visible and adjacent infrared parts of the electromagnetic spectrum, for which the skin is poorly absorbent and particularly reflective. In addition, by changing the voltage across the incandescent filament one changes also the emission spectrum of the electric light bulb. Since the transparency of skin to radiation depends on its wavelength [47], a modification of the emission spectrum influences all parameters of the skin heating process inclusive the volume of tissue affected by the heating. This point is insurmountable when one wishes to vary the intensity of heat stimulation using light bulbs. We used a laser stimulator (CO_2_LSD, SIFEC, Ferrière, Belgium) for the following reasons [4]: (1) it is an infrared monochromatic radiant source with a long wavelength (10.6 µm) for which the absorbance is almost total whatever the pigmentation of the skin and the incidence of the beam; (2) the transparency of skin is weak (∼100 µm), so that the calorific energy absorbed at the level of the cutaneous surface propagates towards nerve endings sensitive to thermal variations, which are localized above the dermo-epidermic junction (60–120 µm depth); (3) the temporal and spatial profile of the calorific energy is well determined; (4) given the high power density of the laser, it is possible to apply abrupt heating. The surface area for stimulation was a circle determined by the Gaussian power profile of the laser beam (See Fig. S1). We chose a diameter of 3.4 mm, for which lateral diffusion of heat by conduction was negligible for at least 2.5 seconds (the longest reaction time recorded in the present study). Beyond this period, diffusion processes gradually and significantly thwart the temperature increase. In the last experiment (Fig. 9C), a 15 mm diameter was used in order to lower the power of the laser beam without such thwarting.

### Monitoring the stimulus

During laser stimulation, the temperature increases proportionally with the square root of time according to the law of radiant heat transfer T = T_0_+a.t^0.5^ (see Fig. 2A and 3A). The constant term a (a^2^ = the slope α of the straight lines in the right graphs of Fig. 2A, 2B & 2B′) is proportional to the power density (Q) of the laser, according to the relation a = K.Q = K.q/S, where K is a composite constant grouping together the biophysical properties of the skin, S the stimulation surface area (mm^2^) and q the laser power (mW).

In practice, for a given surface of stimulation and a constant angle of incidence of the laser beam to the skin, the term a is proportional to q. It follows that α is proportional to q^2^. To reach a satisfactory level of reproducibility, the laser beam must be perpendicular to the stimulated surface because the angle of incidence influences power density. However, the skin is never flat and the tail is a conical cylinder. In order to minimize these sources of variability, the beam axis was targeted perpendicular to the axis of the tail. The beam was adjusted to 45° with respect to the vertical, in order to elicit a contralateral withdrawal movement. Doing this allowed stimulation of the right and left side of the tail at a given rostro-caudal level.

We determined a range of powers of stimulation (100–350 mW) provoking responses within less than 2.5 seconds without damaging the skin. In these conditions, the slope α was in the 0.07–3.2°C^2^/ms range and the maximum temperature reached at the actual moment of the reaction was always lower than 70°C.

### Protocol for stimulation

Four (or 5) sites of stimulation, each 4 (or 3) cm from each other, were identified on each side of the tail. The left and the right side were stimulated alternatively with variable laser powers in the 100–350 mW range. These powers were delivered in a pseudo-random order, 15–20 times per stimulation site. The stimuli were directed successively to the 8 (or 10) sites. A minimum of 5 minutes passed between stimulation of a given site and the next stimulation at the same site. We checked that there were no significant differences between data computed separately from either side of the tail (Lβ: p>0.8; Tβ: p>0.8) or from the two temporal halves of the experiment (Lβ: p>0.3; Tβ: p>0.2).

### Measuring the temperature of the skin

**Figure 10:**
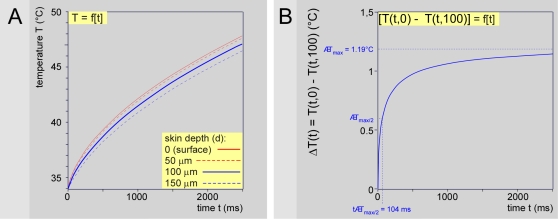
Heat modeling. - A. Simulation of skin temperature at the laser beam axis in time and at different depths d (see insert) using the unidimensional heat conduction differential equation and biophysical parameters of skin described in section Methods with a mid-range laser power density of 25 mW/mm^2^, stimulus duration of 2500 ms and initial skin temperature of 34°C. - B. Temporal evolution of the difference in temperature ΔT at 100 µm skin depth [T(t,100)] and at skin surface [T(t,0)]. The difference grows rapidly towards a plateau of about 1°C. This is the greatest difference as it is obtained at the beam axis.

The measurement of temperature at the skin surface is justified by convenience of use, non-invasive character and possibility of extrapolating the underlying subcutaneous temperatures by modeling. This temperature represents only an approximation of the temperature reached at the level of the nociceptors, which are located at the dermo-epidermal junction, at an average depth of 100 µm [5], [48], [49]. The probes that might be used to make direct measurements (e.g. thermocouples) have not yet been miniaturized to the extent that they would not disturb the thermal field. However, the temperature reached by the various layers within the skin can be estimated through modeling and simulation by means of the unidimensional heat conduction differential equation and biophysical parameters of skin reported in [5], [48], [49], [51], [52]. Figure 10A presents the results of such simulations. It shows the temporal evolution of skin temperature at the surface (d = 0) and at three depths in the skin (d = 50, 100 and 150 µm) using a mid-range laser power density of 0.025 W/mm^2^, stimulus duration of 2500 ms and initial skin temperature of 34°C. Figure 10B shows the temporal evolution of the difference between the temperature at a depth of 100 µm T_d = 100_ and surface skin temperature T_d = 0_, i.e. ΔT_d_ = T_d = 100_−T_d = 0_. It fits a rectangular hyperbola of the form ΔT_d_ = ΔT_max_.t/(t_ΔTmax/2_+t), where ΔT_max_ represents the upper asymptote (i.e. maximum of ΔT) and t_ΔTmax/2_ represents the time t at ΔT_max_/2. The parameters are estimated by linearizing the rectangular hyperbola using the Hans-Woolf procedure [53]:
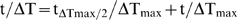
The parameters are computed as follows:
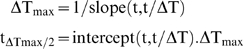
Which for the example given in Figure 10B yields:
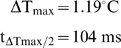



In other words, the difference in temperature between the skin surface and the dermo-epidermal junction at 100 µm depth is about 1°C and half of that difference is reached in about 100 ms. One can consider therefore that the temperature reached at the dermo-epidermal junction in our experiments was slightly lower, but indeed close to the measured surface temperature.

Finally, ΔT_max_ (i.e. maximum of ΔT) increases linearly with laser power density Q. This allows to estimate easily, for a range of laser power densities used in the present experiments, the difference between skin surface temperature and the temperature at a skin depth of 100 µm by means of the following equation:
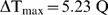



Since we derive the power of emitted laser radiation from the heating ramp by a = K.Q = α^0.5^ where K is a lumped constant equating [2(1−r_10.6_)/(π.k.ρ.c)^0.5^] = 6.564 (see [54] and table 1), the maximum difference in temperature can also be expressed as ΔT_max_ = 5.23 α^0.5^/K.

**Table 1:** Symbols, abbreviations and units. In Text and Figures.

a	α^0.5^ (slope of the heating ramp in °Cs)
α	slope of the squared temperature variation (°C^2^/s) = a^2^
AT	apparent threshold (°C)
CDP	central decision-making process
CNS	central nervous system
Co2	second coccygeal level of the spinal cord
D	distance between the stimulation site and the dorsal horn entry zone (mm) = D_t_+D_c_
D_c_	distance between the tail-trunk interface and the dorsal horn entry zone (mm)
D_t_	distance between the stimulation site and the tail-trunk interface (mm)
ΔAT	temperature variation between the initial temperature and the apparent threshold = AT−T_0_ (°C)
ΔT	temperature variation with reference to the initial temperature = T−T_0_ (°C)
K	composite constant grouping together the biophysical properties of skin
k	thermal conductivity = 0.696 W.m^−1^.°C^−1^
L	latency (ms) = unobserved hypothetical time
Ld	decisional latency (ms) = time required by the CNS for interpreting and processing the nociceptive information
Lm	motor latency (ms) = time from motoneurons activation up to the shortening of the muscle.
Lp	peripheral latency (ms) = transit time for spikes in primary afferent to reach the entry zone in the spinal cord = Lp_t_+Lp_c_
Lp_c_	peripheral latency within the core (ms) = transit time for spikes in primary afferent between the tail-trunk interface and the dorsal horn entry zone
Lp_t_	peripheral latency within the tail (ms) = transit time for spikes in primary afferent between the stimulation site and the tail-trunk interface
Lβ	behavioral latency (ms) = time period which separates the moment at which Tβ is reached from the actual moment of the behavioral response R
Lϕ	physical latency (ms) = duration of the skin heating process from the initial skin temperature T_0_ to trigger transduction in nociceptors
Lτ	transduction latency (ms) = time required for heat to be transduced by nociceptors into neuronal spikes
L2	second lumbar vertebra (or level of the spinal cord)
L4	fourth lumbar vertebra (or level of the spinal cord)
p	absorption coefficient = 20.0 mm^−1^
q	laser power (mW)
Q	density of laser power (mW/mm^2^)
Q_10_	ratio of the conduction velocity at one temperature to the conduction velocity at a temperature 10°C colder.
R	behavioral response
r_10.6_	reflectivity for the wave length of radiation emitted by the C0_2_ laser = 0.78%
ρc	volumetric heat capacity = 4.1868 J.cm^−3^.°C^−1^
S	stimulation surface area (mm^2^)
S2	second sacral level of the spinal cord
t	time (ms)
t_0_	beginning of the stimulation
t_R_	moment of the behavioral response = reaction time (ms)
t_Tβ_	moment when the flow of information generated at the level of the nociceptors is sufficient to trigger a reaction (ms)
T	skin temperature (°C)
T_a_	ambient temperature (°C)
Tβ	behavioral threshold (°C)
T_c_	core temperature (°C)
T_0_	initial skin temperature (°C)
TFL	Tail-flick “latency” = 1000.t_R_ (s)
T8	eighth thoracic vertebra
T10	tenth thoracic vertebra
V	conduction velocity (m/s)
V_c_	conduction velocity of fibers traveling in the core that trigger the behavioral response (m/s)
V_t_	conduction velocity of fibers traveling in the tail that trigger the behavioral response (m/s)

**In Figures:**

Yellow background: related to variable measured experimentally.

Blue background: related to latent variable to be determined.

Brown: related to individual curves of interest.

Although we filmed the full surface area of stimulation, we chose to investigate further the temporal evolution of the warmest pixel. From the physical standpoint, this choice was justified by the Gaussian profile of the beam, reflected by an equivalent spatial profile of the temperature increment (see Fig. S1). Knowing the highest value and the diameter of the temperature profile allows anyone to reconstruct the whole picture.

### The thermometric camera

We used a JADE MWIR (3–5 µm optical bandpass) camera (CEDIP Infrared Systems, Croissy-Beaubourg, France) with a 500 µs integration time, which supplied images of 320×240 pixels at 172 Hz with a sensitivity of 0.02°C at 25°C. It was placed upright to the zone of stimulation and was controlled by the software Cirrus (CEDIP Infrared Systems, Croissy-Beaubourg, France). It was calibrated by means of a black body (CI SR80 CI Systems, Migdal Haemek, Israel). The software Altair (CEDIP Infrared Systems, Croissy-Beaubourg, France) allowed the monitoring of the spatial and temporal evolution of the temperature at the level of the stimulated surface area with 0.3 mm and 5.8 ms resolutions, respectively (See Fig. S2). The recording was triggered 0.2 second before the application of the stimulus.

### Analysis of thermographic films

The analysis of the thermographic films was made by means of the software Altair (CEDIP Infrared Systems, Croissy-Beaubourg, France). This involved the following steps: (1) determination of the zone of interest in the recorded scene; (2) determination of the initial temperature T_0_ in this zone; (3) calculation of the temporal evolution of the warmest pixel in this zone until the image preceding the movement - the ordinate of the ultimate point of this curve constituting the apparent threshold AT. This pixel corresponded to the top of the Gauss curve, which characterizes the spatial profile of the thermal rise (See Fig. S1B). The reaction time t_R_ was measured by counting the number of pictures of the film where the temperature curve exceeded T_0_+1°C and multiplying this count by 1000/172. The analysis of an individual temperature curve included the following steps: (1) transforming the temperature in difference with regard to the initial temperature ΔT = f(t); (2) rising to the square ΔT^2^ = f(t); (3) checking the linearity of this curve; and (4) determining the value of the slope α of this curve.

### Analysis of data brought by a series of tests applied at a given site

At completion of the analysis of the thermographic films related to the tests applied at a given site, a set of temperature curves elicited by a series of laser powers was obtained. Each of these was summarized by the corresponding measured values of T_0_, AT and α the slope of the linear function ΔT^2^ = α.t (see Fig. 2B). Since T_0_ remained stable during the experimental procedure, we could infer the behavioral threshold Tβ and the psychophysical latency Lβ - presumably constant - by two approaches.

#### Method 1: Determination of Tβ and Lβ by changing the time origin

One can modify the representation by adjusting the origin of the time scale of each individual curve for heating to the actual moment of the reaction (See Fig. 2B′). This backward timing procedure is easily performed using the following computation for each individual trial. If x is the negative abscissa of the adjusted graphic (x = t−t_R_), the equation ΔT^2^ = α.t is transformed into ΔT^2^_x_ = α.(x+t_R_). Such a change of origin allows one to visualize the back timing of events and to identify the point (−Lβ on the abscissa and Tβ on the ordinate. Because of the stochastic nature of the psychophysical responses, the points of intersection are distributed in the time vs. temperature plane. It was assumed that the peak value in the bivariate frequency histogram of intersections allowed an estimation of the coordinates −Lβ and Tβ. In practice, the intersections of each curve with all the others were obtained by iterating the method of determinants for solving a system of two linear functions ΔT^2^ (x) vs. x in two unknowns. Density distributions of the coordinates of intersections were computed with bin widths of 20 ms and 0.25°C, respectively. The value corresponding to the peak density of each distribution was considered as an estimate of the point having abscissa −Lβ and ordinate Tβ. It was visualized on a bivariate frequency histogram as shown in Figure 3B and the right panels of Figure 4A.

#### Method 2: Calculation of Tβ and Lβ from relation ΔAT^2^ = f(α)

By substituting α.t_R_ of equation 2 in equation 1, we obtain after rearrangement: ΔAT^2^ = ΔTβ^2^+Lβ.α [**equation 3**]. By plotting ΔAT^2^ as a function of α, the points aligned themselves quite well along a straight line (See Fig. 2C and 3C), the slope and intercept of which corresponding to Lβ and ΔTβ^2^, respectively. Evaluation of the psychophysical latency Lβ and of the behavioral threshold Tβ = T_0_+√ΔTβ^2^ by this method is justified as α is measured independently of ΔAT^2^. In practice, the global analysis of the individual curves from a series of tests included the following steps: (1) building the initial temperature T_0_ histogram; (2) exclusion of data for which T_0_ deviated from the mean by more than two standard deviations; (3) construction of the graph (AT–T_0_)^2^ = f(α); (4) checking the linearity of the function (AT–T_0_)^2^ = f(α); (5) Determination of Tβ and Lβ

### Calculation of the conduction velocity of the fibers that triggered the reaction

Conduction velocities were calculated on the basis of the linear relationship between the distance D from the stimulation site to the dorsal horn entry zone and the corresponding calculated psychophysical latency, Lβ = f(D) = y+D/V, where y is the intercept with the ordinate and 1/V the slope of the straight line.

### Estimations of peripheral (Lp) and decisional (Ld) latencies

Knowing the distance D and the conduction velocity V of the fibers that trigger the reaction, one should be able to estimate the peripheral latency Lp = D/V. However, the conduction velocity of peripheral fibers is highly dependent on temperature, in a way which is equivalent across types, nerves and species [13], [14]. We first measured V along the part of the coccygeal nerve that travels within the tail, maintained at T_0_ = 34°C. But the temperature increases over the length (D_c_) of the coccygeal nerve traveling within the core of the animal, which is maintained at 38°C by thermoregulation processes. Therefore, the decomposition of the peripheral latency into two components (Lp = Lp_t_+Lp_c_) with different conduction velocities (V_t_<V_c_) appeared to be a reasonable assumption (see Fig. S1E). The slope (1/V) of the straight line Lβ = f(D) is reduced in the part of the nerve traveling in the core, with Lp = (D–D_c_)/V_t_+D_c_/V_c_. The intercept y of the straight line with the ordinate of the function Lβ = f(D) is increased by as much : y_c_ = y_t_+D_c_(1/V_t_–1/V_c_). This intercept corresponds to the part of the psychophysical latency that is not devoted to the progress of nociceptive signals along afferent fibers. This is a composite time that includes two successive distinct periods, namely Ld and Lm. Since Lm has been estimated as 4 ms [8], one can finally estimate Ld as y_c_–4. In summary, both Lp and Ld can be estimated if one knows the relative contribution of the afferent path within the core of the animal, which means estimation of D_c_ and V_c_.

### Data related to the afferent path within the core of the animal

Estimation of both D_c_ and V_c_ is possible if data related to various initial temperatures of the skin T_0_ are available. The experiments described above were replicated with the rat being introduced to a chamber where the ambient temperature T_a_ was maintained stable during a given session, but changed over sessions in the 17–34°C range. The tail was not intentionally heated and was stimulated at 3–4 rostro-caudal levels. The temperature of the tail T_0_ was slightly above ambient temperature T_a_ for T_a_≤25°C, and several degrees above for T_a_≥32°C (See Fig. 7A). Between 25 and 30°C, the basal temperature was essentially variable, the vasomotor tone of the tail oscillating over time between vasoconstriction and vasodilatation phases (See Supporting Video S1). In these later cases, the behavioral tests were performed during vasodilatation phases. The results from this study revealed a very significant V_t_ = f(T_0_) linear function best described by the equation V_t_ = 0.041 T_0_–0.471, which provided the possibility of calculating the conduction velocities of fibers at core temperature T_c_: V_c_ = V_t_+0.041 (T_c_–T_0_). The V_t_ to V_c_ change, which occurs at the tail-core interface where the temperature of the nerves increases from T_0_ to the core temperature T_c_, can be estimated statistically in the D-Lβ plot by considering the overall cluster of the intersection points of each straight line with the others (Fig. 7D). A crossed tabulation of the slope and intercept of each straight line was used to compute the median values of the coordinates of these intersection points, which are estimations of D_c_ = length of the coccygeal nerve traveling within the trunk at core temperature and (Lp_c_+Ld+Lm), respectively.

### Statistical analyses

Least squares linear regressions and one-way analyses of variance (ANOVA) were used for statistical purposes. Calculations were performed with the statistical software Staview™ 5.0 and Statgraphics™ Plus 5. Other calculations were made with the software Mathcad™ or Matlab™. Results were considered significant at P<0.05. Data are expressed as means (±confident interval 95%).

## Supporting Information

Figure S1 Analysis of thermal imaging of the skin during CO_2_ laser stimulation. A. Examples of thermal imaging of the skin during CO_2_ laser stimulation. The thermometric camera provided a picture every 5.8 ms (sampling rate 172 Hz). A sample of 5 pictures is shown, filmed 58, 122, 103, 308, and 477 ms following the triggering of the stimulus (a–e, from bottom to top; color temperature scale on the left; resolution 0.3 mm). The edges of the tail of the animal are indicated by the white dotted lines. Image e was recorded just before the withdrawal response - B. Spatial profiles of the pictures shown in A. They were calculated on the rostro-caudal line passing through the center of the stimulation spot. Note they all fit a Gaussian curve. One can compare the radius of the stimulation spot with the radius of the laser beam. The radius of the beam is defined as the distance separating its z axis from the zone where its power is reduced to 1/e^2^ = 13.5% of its maximum. A corresponding radius of the stimulation spot resulted from these properties of the beam: the distance separating the z-axis of the heating spot from the zone where the difference of temperature is reduced to 13.5% of the maximum was 1.7 mm. The physical diameter of stimulation (3.4 mm) is shown as a yellow area. - C. Temporal evolution of the temperature of the warmest pixel during this laser stimulation. AT = apparent threshold; R = behavioral response; t_R_ = reaction time.
(1.50 MB TIF)

Figure S2 Individual heating curves corresponding to the example analyzed in Fig. 6. The tail was stimulated at three rostro-caudal levels (dark to light blue curves from top to bottom; distance D that separated the site of stimulation on the tail from the entry zone in the cord is indicated below curves) in three different ambient temperatures that maintained the mean T_0_ at 18.5 (A, blue), 30.9 (B, green) and 36.0°C (C, red), respectively. Note the clear tendency of these curves to cross each other in a privileged zone (open circles) and the progressive shift of this zone both backward in time when the stimulation site moved from proximal to distal parts of the tail and upward when the ambient temperature increased (dashed lines).
(1.55 MB TIF)

Figure S3 Modeling and simulation of the tail-flick. Equation 1 can be written: t_R_ = f(D) = (Tβ−T_0_)^2^/α+(D−90)/V_t_+90/V_c_+Ld+Lm = (Tβ−T_0_)^2^/α+(D−90)/V_t_+90/[V_t_+0.041 (T_c_−T_0_)]+Ld+Lm. This opens the possibility of computing variations of t_R_ introduced by moving the stimulation site along the tail. The graphs shows results of such a procedure, with the numerical values determined from the present study with the basal temperature of the tail stabilized at 34°C. The numerical values of α was chosen as 0.045°C^2^/s in order to achieve the 2–4 seconds range, shown as a yellow area, for the TFL commonly reported in the literature for the classical tail-flick test in control situations [35]. The other numerical values were: T_c_ = 38°C, T_0_ = 34°C, V_t_ = 0.91 (0.81–1.01) m/s, Ld = 132 (117–146) ms, Lm = 4 ms. The results of computation are shown as means ±95% C.I. (black, red and blue curves, respectively). - A. The behavioral threshold Tβ is considered as invariant (value indicated in insert). In those cases, the TFL increases slightly when the stimulation site moves distally along the tail, as one would intuitively expect. - B. Since there was a significant proximo-distal shift of Tβ (Fig. 5B), we introduced this factor of variation in the model (relation Tβ = f(D) indicated in insert), transforming the linear function to a quadratic relationship. This modification skewed the D-t_R_ relationship in such a way that t_R_ was shortened when the site of stimulation moved distally. In other words, the model predicts a negative correlation between the rostro-caudal position of the stimulation site and the tail-flick “latency” (at least when α<0.13), even though the pathway for the afferent signals increases. In fact, this a priori paradoxical property has already been described [20], [37], which a posteriori supports the present model.
(2.31 MB TIF)

Video S1 A. An example of thermographic film of the tail of a rat placed in a conventional restrainer, recorded with a 320×240 pixels resolution at 1 Hz during 135 minutes. Steel rings maintained its tail. - B. Corresponding temporal evolution of the skin temperature recorded on 6 points distributed from the base to the tip of the tail (blue dots and corresponding traces). One observes phases of vasoconstriction and vasodilatation throughout the recording session. Note that the 10°C amplitude of the variations for the most distal points of the tail. As measured on an inert piece of wood (Ta, lower brown trace), the ambient temperature was stable around 25°C. The central core temperature (Tc) is shown as upper black trace. - C. The dissipation of heat is regulated by abrupt variations of blood flow in a system of arterio-venous anastomoses, which form a double ladder. Anatomy adapted from [58].
(12.19 MB MOV)
