# Supplementary figures and images for: Peripheral and Central Determinants of a Nociceptive Reaction: An Approach to Psychophysics in the Rat
Source: PLoS One. 2008 Sep 3;3(9):e3125. doi: 10.1371/journal.pone.0003125 (PMC2518957; doi:10.1371/journal.pone.0003125)

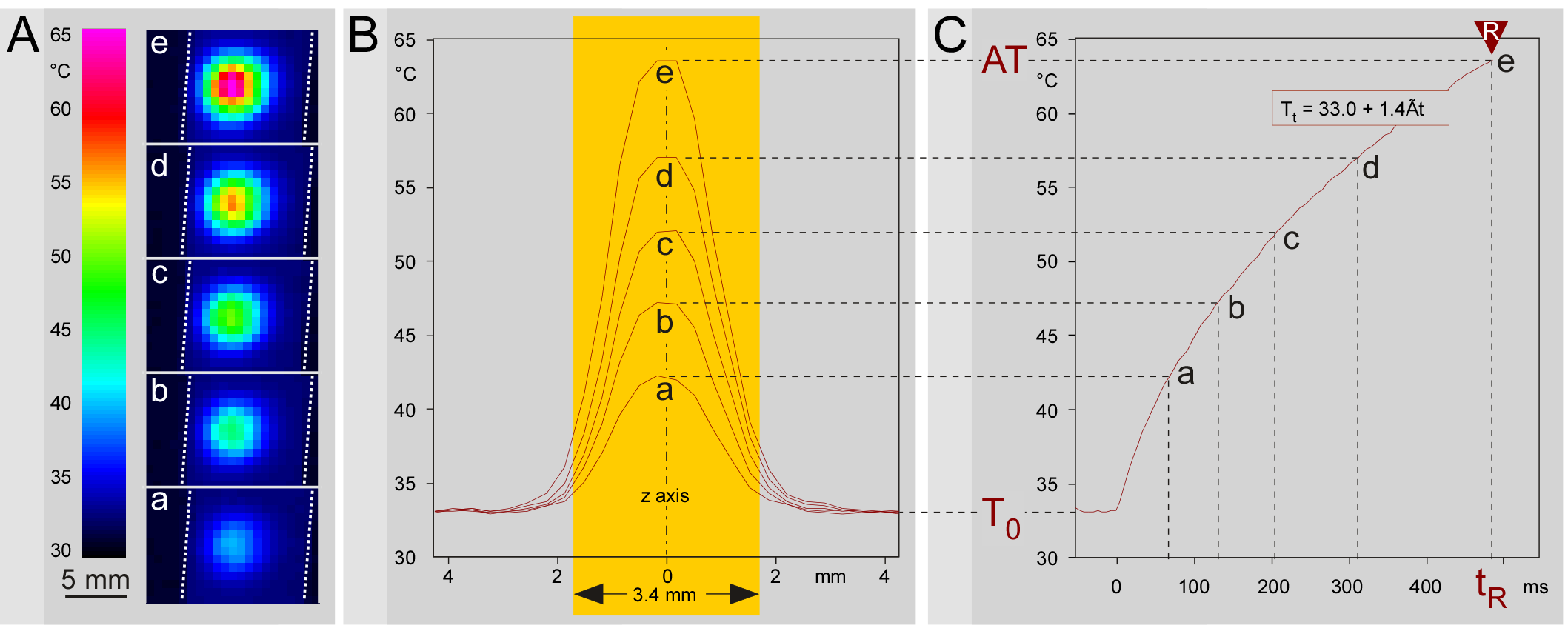

Supplement: Figure S1 — Analysis of thermal imaging of the skin during CO2 laser stimulation. A. Examples of thermal imaging of the skin during CO2 laser stimulation. The thermometric camera provided a picture every 5.8 ms (sampling rate 172 Hz). A sample of 5 pictures is shown, filmed 58, 122, 103, 308, and 477 ms following the triggering of the stimulus (a–e, from bottom to top; color temperature scale on the left; resolution 0.3 mm). The edges of the tail of the animal are indicated by the white dotted lines. Image e was recorded just before the withdrawal response - B. Spatial profiles of the pictures shown in A. They were calculated on the rostro-caudal line passing through the center of the stimulation spot. Note they all fit a Gaussian curve. One can compare the radius of the stimulation spot with the radius of the laser beam. The radius of the beam is defined as the distance separating its z axis from the zone where its power is reduced to 1/e2 = 13.5% of its maximum. A corresponding radius of the stimulation spot resulted from these properties of the beam: the distance separating the z-axis of the heating spot from the zone where the difference of temperature is reduced to 13.5% of the maximum was 1.7 mm. The physical diameter of stimulation (3.4 mm) is shown as a yellow area. - C. Temporal evolution of the temperature of the warmest pixel during this laser stimulation. AT = apparent threshold; R = behavioral response; tR = reaction time. (1.50 MB TIF) [file pone.0003125.s001.tif]

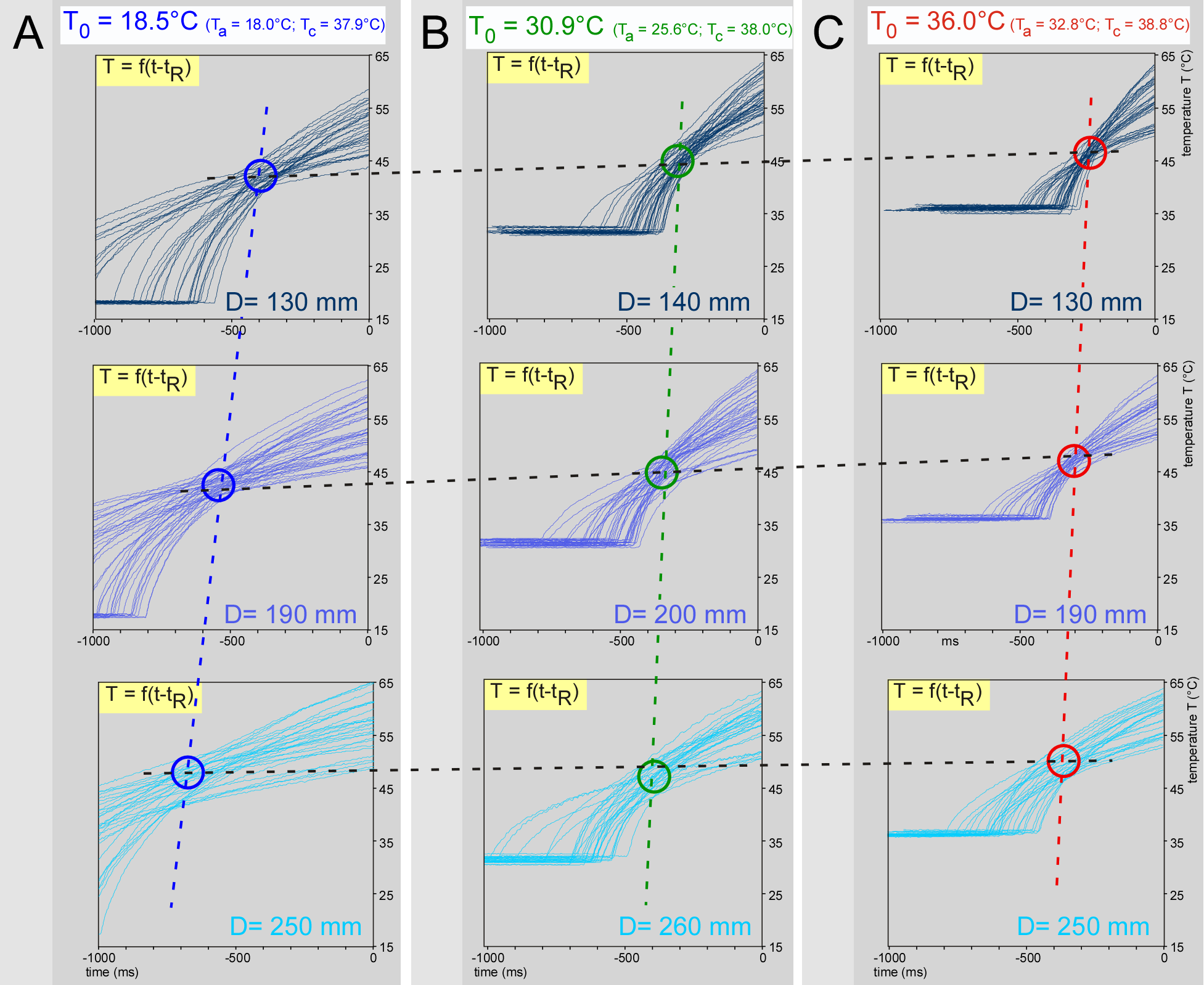

Supplement: Figure S2 — Individual heating curves corresponding to the example analyzed in Fig. 6. The tail was stimulated at three rostro-caudal levels (dark to light blue curves from top to bottom; distance D that separated the site of stimulation on the tail from the entry zone in the cord is indicated below curves) in three different ambient temperatures that maintained the mean T0 at 18.5 (A, blue), 30.9 (B, green) and 36.0°C (C, red), respectively. Note the clear tendency of these curves to cross each other in a privileged zone (open circles) and the progressive shift of this zone both backward in time when the stimulation site moved from proximal to distal parts of the tail and upward when the ambient temperature increased (dashed lines). (1.55 MB TIF) [file pone.0003125.s002.tif]

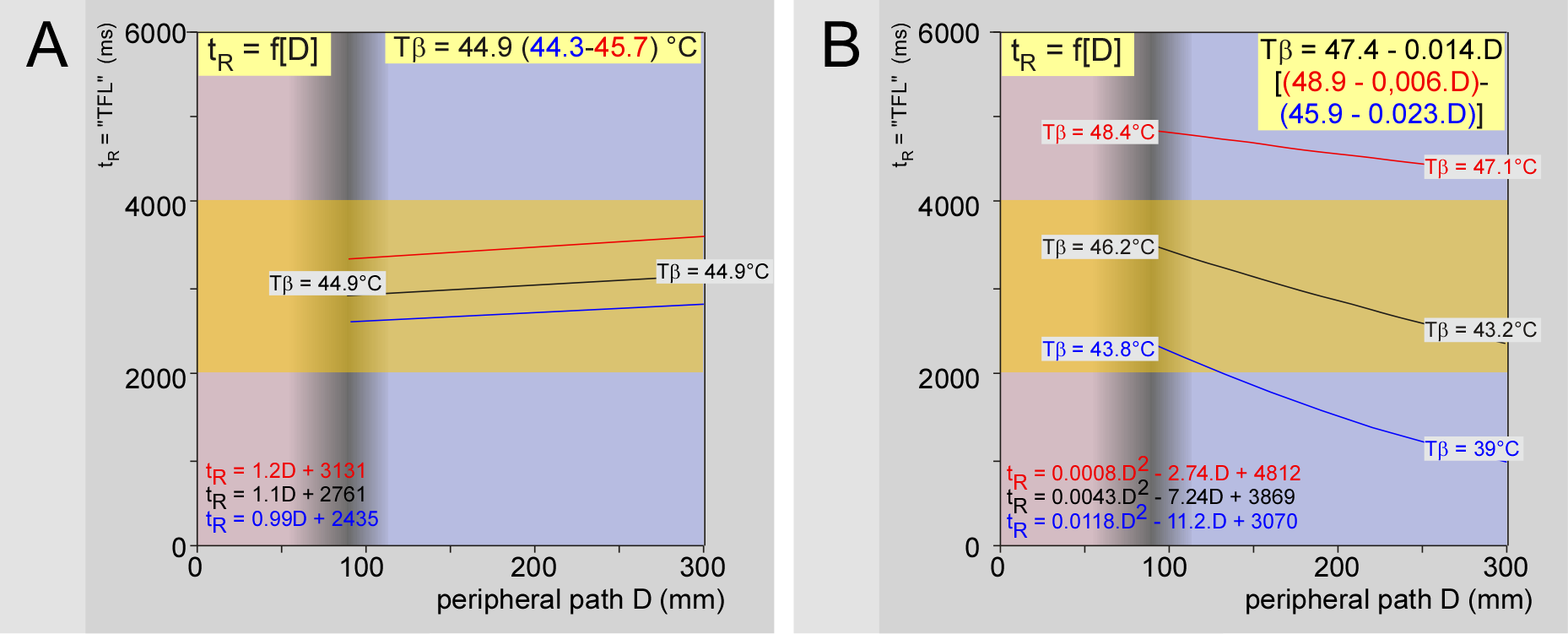

Supplement: Figure S3 — Modeling and simulation of the tail-flick. Equation 1 can be written: tR = f(D) = (Tβ−T0)2/α+(D−90)/Vt+90/Vc+Ld+Lm = (Tβ−T0)2/α+(D−90)/Vt+90/[Vt+0.041 (Tc−T0)]+Ld+Lm. This opens the possibility of computing variations of tR introduced by moving the stimulation site along the tail. The graphs shows results of such a procedure, with the numerical values determined from the present study with the basal temperature of the tail stabilized at 34°C. The numerical values of α was chosen as 0.045°C2/s in order to achieve the 2–4 seconds range, shown as a yellow area, for the TFL commonly reported in the literature for the classical tail-flick test in control situations [35]. The other numerical values were: Tc = 38°C, T0 = 34°C, Vt = 0.91 (0.81–1.01) m/s, Ld = 132 (117–146) ms, Lm = 4 ms. The results of computation are shown as means ±95% C.I. (black, red and blue curves, respectively). - A. The behavioral threshold Tβ is considered as invariant (value indicated in insert). In those cases, the TFL increases slightly when the stimulation site moves distally along the tail, as one would intuitively expect. - B. Since there was a significant proximo-distal shift of Tβ (Fig. 5B), we introduced this factor of variation in the model (relation Tβ = f(D) indicated in insert), transforming the linear function to a quadratic relationship. This modification skewed the D-tR relationship in such a way that tR was shortened when the site of stimulation moved distally. In other words, the model predicts a negative correlation between the rostro-caudal position of the stimulation site and the tail-flick “latency” (at least when α<0.13), even though the pathway for the afferent signals increases. In fact, this a priori paradoxical property has already been described [20], [37], which a posteriori supports the present model. (2.31 MB TIF) [file pone.0003125.s003.tif]
